# Inclusion of Polypills for Prevention of Cardiovascular Disease in the 23rd World Health Organization Model List of Essential Medicines: A Significant Step Towards Reducing Global Cardiovascular Morbidity and Mortality

**DOI:** 10.5334/gh.1310

**Published:** 2024-02-26

**Authors:** Anubha Agarwal, Mark D. Huffman

**Affiliations:** 1 Department of Medicine and Global Health Center, Washington University in St. Louis, St. Louis, MO, US; 2 The George Institute for Global Health, University of New South Wales, Sydney, AU

**Keywords:** Cardiovascular disease, polypills, World Health Organization, essential medicines

## Abstract

This commentary describes the potential impact of inclusion of polypills for prevention of cardiovascular disease in the 23rd WHO Model List of Essential Medicines, and provides a roadmap for adoption, implementation, sustainment, and scale-up. The World Health Organization’s endorsement of polypills is essential for improving global access, particularly in low- and middle-income countries. The greatest health gains are expected in a primary prevention population which has a significantly higher burden of fatal and non-fatal cardiovascular disease compared with the population of individuals with prevalent cardiovascular disease. A focus on adoption, implementation, sustainment, and scale-up of polypills for prevention of cardiovascular disease is needed including increasing supply of available polypills and incorporating polypills into the World Health Organization HEARTS technical package for integration into primary care systems to realize these benefits for population health. Widespread implementation of polypills for prevention of cardiovascular disease has the potential to equitably reduce the impact of cardiovascular disease globally by simplifying treatment options and expanding accessibility across economic levels, both across and within countries.

## Introduction

The concept of a ‘polypill’ for cardiovascular disease targeting multiple modifiable risk factors was first introduced in 2001 by Professor Richard Peto and gained wider recognition in 2003 in a seminal modeling paper by Professors Wald and Law as a population-level strategy to reduce cardiovascular disease by more than 80% if taken by all individuals aged 55 years and older [1]. Two decades later, in 2023, the scientific understanding of polypills has advanced considerably from concept to evidence from randomized clinical trials resulting in a landmark decision by the World Health Organization (WHO) to include polypills for high-risk primary and secondary prevention of cardiovascular disease in the 23rd Model List of Essential Medicines [2]. Based on a systematic review synthesized in the application to the WHO Essential Medicines List, polypills with at least one blood-pressure lowering drug and one lipid-lowering drug are associated with a lower risk of all-cause mortality by 11% (5.6% versus 6.3%, RR = 0.89, 95% CI 0.78, 1.00, 4 trials, 9,327 participants) and fatal or non-fatal atherosclerotic cardiovascular disease events by 29% (6.1% versus 8.4%, RR = 0.71, 95% CI 0.63, 0.79, 5 trials, 15,503 participants) in a primary prevention population at increased risk of cardiovascular disease. Even greater effects are anticipated in populations with lower baseline treatment rates than observed in these trials, which represents most patients around the world [3]. One adequately powered trial (SECURE) in a secondary prevention population with prevalent atherosclerotic cardiovascular disease demonstrated polypills reduced the risk of major adverse cardiovascular events by 24% compared to a well-treated usual care group (9.5% versus 12.7%, HR = 0.76, 95% CI 0.60, 0.96) [4]. The essential question now is how do we translate these effect sizes to achieve global targets set by the United Nations of reducing the risk of premature mortality from noncommunicable diseases by 2030? This commentary describes the potential impact of inclusion of polypills for prevention of cardiovascular disease in the 23rd WHO Model List of Essential Medicines, and provides a roadmap for adoption, implementation, sustainment, and scale-up.

## Potential Impact

The potential impact of inclusion of polypills for prevention of cardiovascular disease in the latest WHO Essential Medicines List in reducing global cardiovascular morbidity and mortality is profound. The greatest gains are expected in a primary prevention population which has a significantly higher burden of fatal and non-fatal cardiovascular disease compared with the population of individuals with prevalent cardiovascular disease. Reductions in non-fatal cardiovascular disease events, including myocardial infarctions and strokes, are estimated to be 3–4-fold greater than reductions in cardiovascular mortality alone based on the distribution of cardiovascular disease events estimated by the Global Burden of Disease and Prospective Urban Rural Epidemiology Study [5,6]. Thus, widespread implementation of polypills could have major public health gains, even beyond achieving global targets in the United Nations Sustainable Development Goals. The WHO’s endorsement of polypills for cardiovascular disease prevention is essential for improving global access, particularly in low- and middle-income countries where undertreated, at-risk, individuals may have the most to gain from polypill pharmacotherapy. The WHO Essential Medicines List is used by member states to guide their national Essential Medicines List which affects drug procurement, availability, and affordability through tariff exemption and prequalification in the public sector [7]. Furthermore, WHO’s endorsement may enhance investments in polypills for cardiovascular disease prevention, may make national governments and international agencies more willing to pay for polypills in health insurance benefits packages, and facilitate health care workers to prescribe polypills.

## Roadmap for Adoption, Implementation, Sustainment, and Scale-Up

Although the inclusion of polypills on the WHO Essential Medicines List is a landmark decision, it will not be a panacea to stem the global cardiovascular disease epidemic without a focus on adoption, implementation, sustainment, and scale-up. Training of healthcare workers on polypill-based clinical algorithms including use of polypills as essential background therapy, alignment of polypill use with other tasks for prevention of cardiovascular disease, and incorporation in clinical guidelines will help increase adoption of cardiovascular polypills. Implementation, sustainment, and scale-up of polypills for prevention of cardiovascular disease requires a broader view of health system constraints limiting current use including the supply chain, quality, and access to polypills. The current supply of available polypills for prevention of cardiovascular disease with marketing authorization is insufficient to meet the current and projected demand. Creative incentives are needed for large-scale pharmaceutical companies to invest in manufacturing polypills such as advanced bulk purchasing by governments and international agencies to mitigate financial risk, streamlined paths to regulatory approval for fixed-dose combination polypills with proven benefits, and patent protections. Pricing models will need to ensure polypills are affordable to be implemented at scale to improve population health. Once available and affordable, polypills should be integrated into the WHO HEARTS technical package for integration into primary care systems as a path to sustainment and scale-up.

## Conclusion

Wide-spread implementation of polypills for prevention of cardiovascular disease has the potential to equitably reduce the impact of cardiovascular disease globally by simplifying treatment options and expanding accessibility across economic levels, both across and within countries. It is time to use collective sociopolitical capital in academia, industry, and government sectors to adopt, implement, sustain, and scale polypills for prevention of cardiovascular disease to maximize public health benefit and stem the rising threat of cardiovascular disease globally.
